# Custom-made implants for massive acetabular bone loss: accuracy with CT assessment

**DOI:** 10.1186/s13018-023-04230-5

**Published:** 2023-09-30

**Authors:** Matteo Romagnoli, Marco Zaffagnini, Eleonora Carillo, Federico Raggi, Marco Casali, Alberto Leardini, Giulio Maria Marcheggiani Muccioli, Alberto Grassi, Stefano Zaffagnini

**Affiliations:** 1 Ortopedia e Traumatologia Rizzoli Argenta, Via Nazionale Ponente 5, 44011 Argenta, FE Italy; 2 Clinica Ortopedica e Traumatologica 2, IRCCS Istituto Ortopedico Rizzoli, Via Pupilli 1, 40136 Bologna, BO Italy; 3 Movement Analysis Laboratory, IRCCS Istituto Ortopedico Rizzoli, Via di Barbiano, 1/10, 40136 Bologna, Italy

**Keywords:** Custom-made, Hip, Arthroplasty, Bone loss, Revision total hip arthroplasty (rTHA), Acetabular revision, Patient-specific, Accuracy, Positioning, Computer tomography

## Abstract

**Background:**

Custom-made implants are a valid option in revision total hip arthroplasty to address massive acetabular bone loss. The aim of this study was to assess the accuracy of custom-made acetabular implants between preoperative planning and postoperative positioning using CT scans.

**Methods:**

In a retrospective analysis, three patients who underwent an acetabular custom-made prosthesis were identified. The custom-made designs were planned through 3D CT analysis considering surgical points of attention. The accuracy of intended implants positioning was assessed by comparing pre- and postoperative CT analyzing the center of rotation (CoR), anteversion, inclination, screws, and implant surface in contact with the bone.

**Results:**

The three cases presented satisfactory accuracy in positioning. A malpositioning in the third case was observed due to the posterization of the CoR of the implant of more than 10 mm. The other CoR vectors considered in the third patient and all vectors in the other two cases fall within 10 mm. All the cases were positioned with a difference of less than 10° of anteversion and inclination with respect to the planning.

**Conclusions:**

The current case series revealed promising accuracy in the positioning of custom-made acetabular prosthesis comparing the planned implant in preoperative CT with postoperative CT.

**Supplementary Information:**

The online version contains supplementary material available at 10.1186/s13018-023-04230-5.

## Introduction

The estimated annual volume of total hip arthroplasty (THA) is estimated to increase significantly in the next decades [1]. Despite continuous improvement in implant materials and design, the amount of revision THA (rTHA) will expect to grow too, increasing by 19% in 2050 [2]. At a 10-year follow-up, the revision rate of THA of 12% is to be expected [3]. Revision of the acetabular component is a challenging surgery in orthopedics, particularly in massive acetabular bone defects, despite the recent algorithms and tools for large bone removal and replacement in the pelvis [4, 5]. In clinical practice, several types of bone grafting like impaction bone grafting, autografts, allografts, and synthetic bone grafts are largely used to fill the acetabular bone loss [6–8]. However, bone grafts may not always be enough to obtain satisfactory fixation, thus different types of revision implants have been developed [9, 10]. The most successful acetabular implants used are hemispheric acetabular components [11], cages [12], oblong components [13], iliac screw cups [14], augments [15], modular acetabular systems, or acetabular custom-made implants [16–18]. In the literature, acetabular bone defects were described widely with the Paprosky’s classification, shown to have high validity and reliability, where types 3A and 3B represent the most severe forms of massive bone defects [19, 20]. In particular, type 3A defect includes moderate lysis of the medial wall, severe superolateral migration, i.e., > 2 cm, of the superior dome, intact anterior column, and moderate lysis of the posterior column, whereas type 3B defect includes severe lysis of the medial wall, severe superomedial migration, i.e., > 2 cm, of the superior dome, disrupted anterior column, and severe lysis of the posterior column [19].

For the management of severe periacetabular bone deficiency in types 3A and 3B of Paprosky’s classification, a custom-made implant might be the best alternative [10]. However, the accuracy of the positioning of the custom-made implant might be compromised from the intra-operative situation, so is necessary to assess the reproducibility of the preoperatory planning. The use of computed tomography (CT) scans permits a comparison between the planning and the postoperative positioning of the implant, thus verifying the accuracy and reliability of the use of those innovative technologies. Literature shows few results regarding the accuracy of the restoration of the center of rotation (CoR), inclination (INCL), and anteversion (AV) of the custom-made implants demonstrating encouraging outcomes [21–25]. To the best of the authors’ knowledge, no study so far has analyzed the percentage of custom-made implants surface in contact with the bone, a requirement to obtain sufficient primary stability [26].

The aim of this case series was to assess the accuracy of three custom-made acetabular implants between preoperative planning and postoperative position using 3D CT in acetabular Paprosky type 3 defects.

## Methods

### Study design

The current study is a retrospective analysis of three acetabular revisions performed between 2019 and 2022. All surgery procedures were performed by one senior surgeon (MR) at Istituto Ortopedico Rizzoli (Bologna, BO, Italy), or Ospedale Mazzolani Vandini Rizzoli-Argenta (Argenta, FE, Italy). All the patients were suitable candidates to receive custom-made 3D printed acetabular cups, by the Lima ProMade© (Lima Corporate, San Daniele del Friuli, UD, Italy).

### Patient details

A total of three patients underwent an acetabular revision with custom-made implants. Patients included were two females aged 57 and 73 years old, and one man, aged 73 years old. The median BMI of the patients was 25.9 kg/m^2^ (range 25–27.4). Aseptic loosening, failure of the previous implant, and multiple revision history for infection were the surgery indication. Concerning acetabular bone loss, a Paprosky 3B with pelvic discontinuity was the type in one case, and a Paprosky 3A in the other two patients.

### Implant design and postoperative management

Before surgical time, a 3D CT analysis of the entire pelvis and femoral implant was performed to determine surgical points of attention. Bone quality, broken screws, the extent of acetabular osseous defect, and integration between acetabular components with the patient’s bone structure were the critical points considered.

Based on CT scans, the custom implant was designed taking into consideration the ideal CoR, optimal implant INCL, AV, and percentage of implant surface in contact with the bone. The CoR was detected by overlapping the 3D reconstruction to the X-ray image; otherwise, anatomical landmarks were used. The position of the CoR was decomposed into three different orthogonal components: anteroposterior (AP), mediolateral (ML), and craniocaudal (CC). Comparing preoperative and postoperative CTs, a cranial, lateral, or posterior shift expressed in millimeters was evaluated.

Designing the custom implant involved a number of steps. Among these, an accurate shape of the acetabular component, primary fixation with iliac and ischial screws, an optimal position of the CoR, and a correct INCL and AV were planned. To achieve these goals, a close collaboration between surgeons and engineers was required. The mean time of implant design and manufacturing, after the final approval of a digital version by the surgeons and the hospital’s administration, has been around 4 weeks.

The diagnosis of periprosthetic joint infection (PJI) was ruled out both preoperatively and intra-operatively for all patients. Before surgery, C reactive protein and erythrocyte sedimentation rates were analyzed for 2 months every 2 weeks, to rule out the presence of possible active infections. Moreover, leukocyte scintigraphy was obtained. During surgery, an analysis of fresh sections of periprosthetic tissue to verify real-time leucocytes was carried out. Meanwhile, in all cases during the surgery, specimens of periprosthetic tissue were collected and sent to microbiology.

All surgeries were performed by a single senior surgeon (M.R.), an experienced specialist in hip surgery. Implantation procedures were ruled out without patient-specific instrumentation and without drill guides. Consequently, screws preparation and fixation were performed with free-hand technique. However, 3D plastic anatomical models of the acetabular and implants were used intra-operatively to support an appropriate implant placement.

For all cases, following the surgery, the hip was placed for 1 month in a hip brace, fixed at 25° of abduction and in neutral rotation, and unlocked up to 70° of flexion and 10° of extension to allow the flexion exercises, commencing from the first day after surgery. No weight bearing was allowed for the first month.

### Postoperative analysis

A postoperative CT scan for all patients was performed. Analyzing preoperative and postoperative CT scans a comparison of the CoR, AV, INCL, screws number, and implant surface in contact with the bone planned vs achieved was ruled out.

## Results

Demographics and implant accuracy positioning data including the difference between planned and achieved CoR positioning, INCL, AV and percentage of surface in contact with the bone are summarized in Table 1. The patients underwent radiological and clinical follow-up to the last one at 4 years, 2 years, and 2 months, respectively. No re-revision, dislocation, infection, or fracture occurred at the last follow-up. None of the patients included in the study has been reoperated.

**Table 1:** Case results

Patient ID	Age (year)	BMI	CoR (posterior shift) (mm)	CoR (lateral shift) (mm)	CoR (cranial shift) (mm)	ΔINCL	ΔAV	Percentage of surface in contact with the bone (%)
Case 1	73	27.4	+ 1	+ 7	− 2	+ 0.7°	− 4.5°	74.1
Case 2	57	25	+ 2.2	+ 6.3	− 0.7	− 0.2°	+ 4.7°	27.6
Case 3	73	25.4	+ 14.8	+ 6.7	+ 10	− 5°	− 6°	61.5

*BMI* body mass index, *y* years, *AV* anteversion, *CoR* center of rotation, *INCL* inclination

### Case 1

The first patient was a female, BMI of 27.4 kg/m^2^, with a strongly reduced activity level. A THA to treat osteoarthritis of the right hip had been performed. The preoperative analysis identified a Paprosky 3B acetabular defect with a pelvic discontinuity. The custom-made implant was designed from a CT scan of the entire pelvis and proximal femurs performed 6 months prior to the operation.

**Fig. 1:**
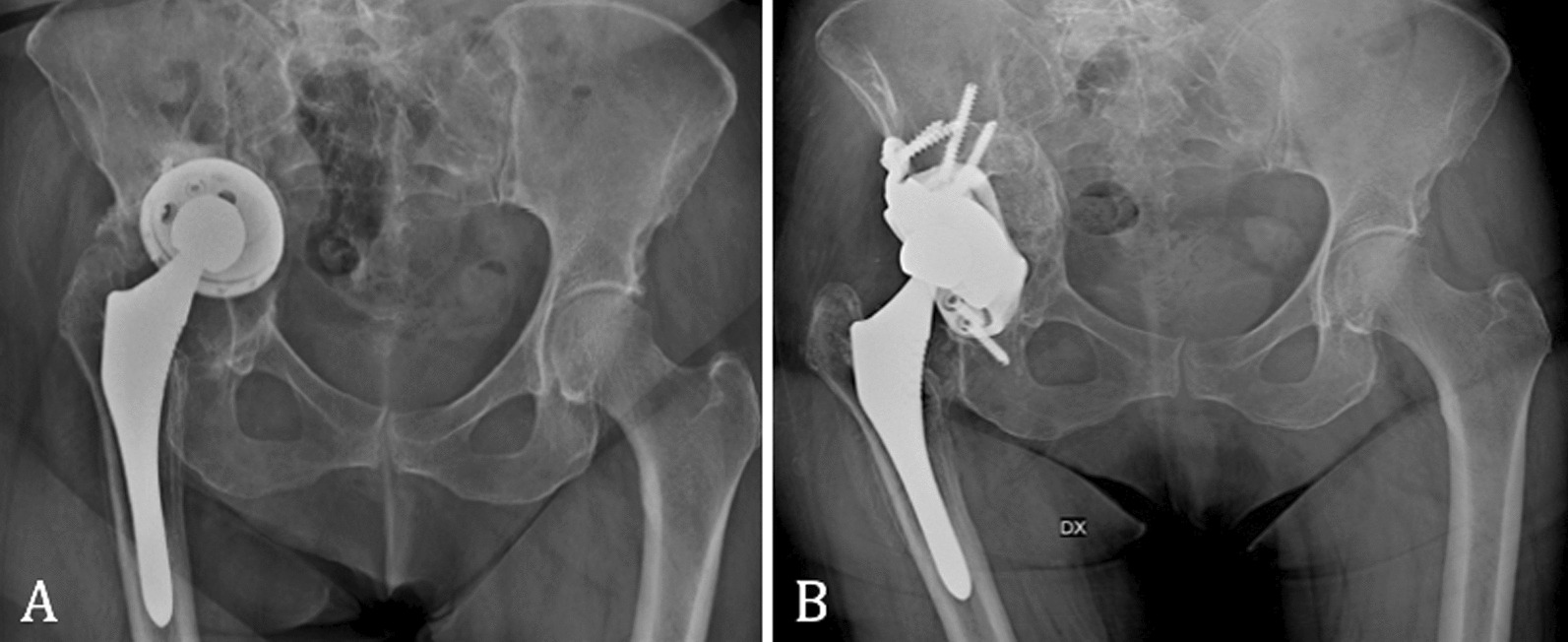
**A** Pre- and **B** post-custom-made implant

Following aseptic mobilization for polyethylene wear, an acetabular custom-made implant through an anterior approach by Enneking was ruled out (Fig. 1).

A double flange implant with four iliac screws and two ischial screws was designed taking into consideration the observed poor bone quality, the disruption and medial protrusion of the acetabular medial wall, the missing anterior wall, and the medial neo acetabulum integrated with the cup in situ. The CoR was detected by using an implant superimposition obtained by iliac alignment.

3D anatomical plastic sterile models were utilized to support the necessary comparison of the acetabular exposition and the corresponding prepared model. Homologous bone grafts in chips and Cerasorb^®^ were used to fill the massive medial and anterior wall defects, then the custom-made prosthesis was implanted.

**Fig. 2:**
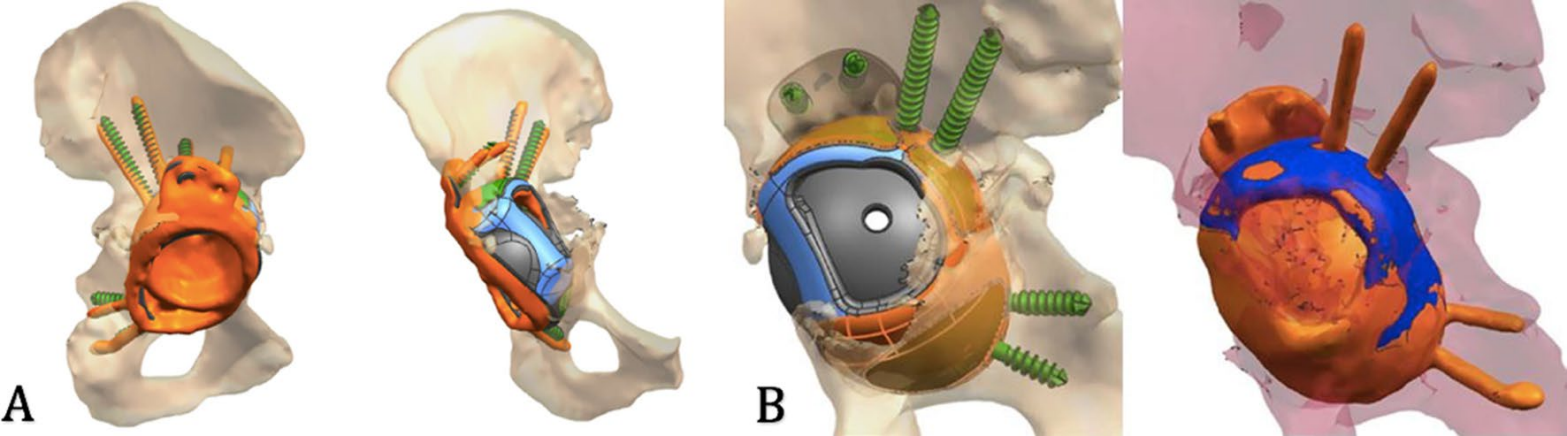
**A** Planned implant (green) versus achieved positioning (orange); **B** trabecular structure in contact with the bone, planned (orange) vs achieved (blue)

The achieved INCL position of the implant was 40° compared to the 45° planned, while the achieved AV was 25.7° compared to the 25° planned. The percentage between the planned and achieved implant surface in contact with the bone was 74.1 %, as the planning resulted in a trabecular surface of 4824 mm^2^, while the achieved one was 3577 mm^2^ (Fig. 2).

Finally, all the six screws were used and oriented in the planned direction. As regards the length of the screws, two out of the four iliac screws were as long as planned, two iliac screws turned out to be longer than the planning, one ischial screw turned out to be longer than the planning, and another one was as long as planned (Additional file 1).

### Case 2

**Fig. 3:**
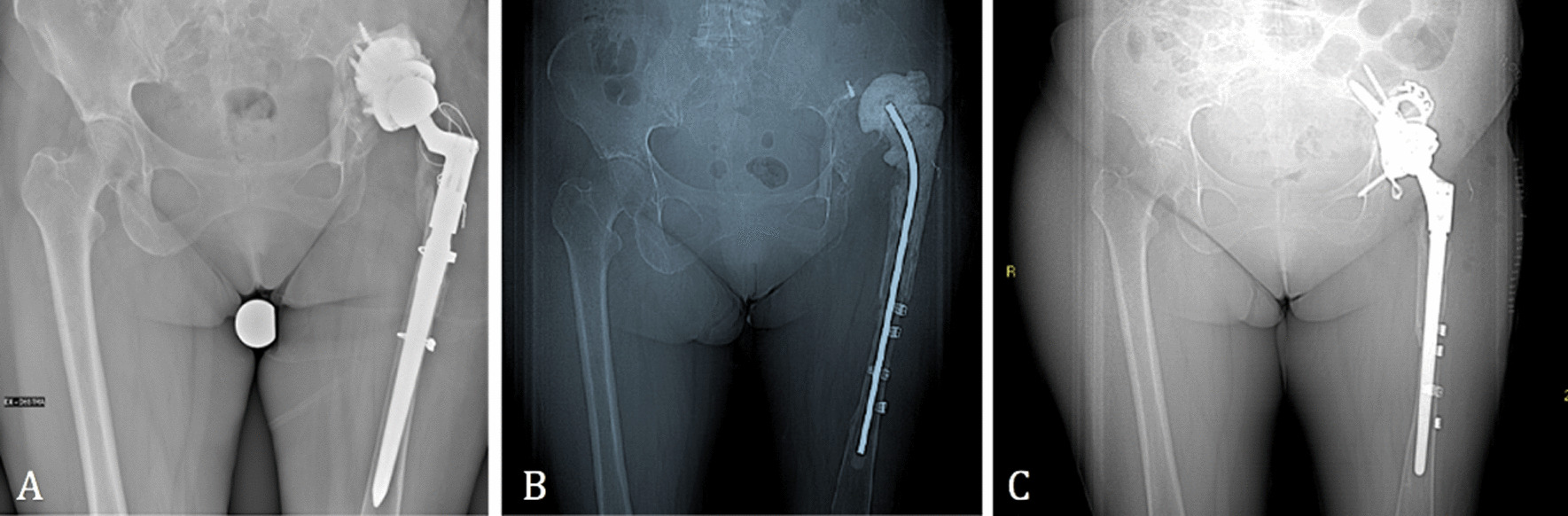
**A** Prosthesis implant before cement spacer, **B** pre- and **C** post-op with a custom-made implant

The second patient was a female, with a BMI of 25 kg/m^2^, who had a history of multiple hip surgeries to treat congenital dysplasia of the left hip. The custom-made implant was designed from a CT scan performed 24 months prior to the operation. A methicillin-resistant staphylococcus epidermidis (MRSE) prosthesis infection of the revision implant occurred, and a two-stage revision was thus performed. In the first stage, an antibiotic-loaded cement spacer was positioned, and a Paprosky 3A acetabular defect was identified. Afterward, in the second stage, the custom-made prosthesis through a posterolateral approach was implanted (Fig. 3).

A double flange implant with four iliac screws, two ischial screw, and one iliac stem was designed taking into consideration the poor bone quality, a screw fragment in the ilium that had to be avoided in screw positioning, and a poor CT scan quality leading to uncertainty in 3D reconstruction.

3D anatomical plastic sterile models were utilized to support the necessary comparison of the acetabular exposition and the corresponding prepared model. Homologous bone grafts in chips and Cerasorb^®^ were used to fill the massive medial wall defect, then the custom-made prosthesis was implanted. Moreover, to minimize the dislocation risk, a double mobility insert was used. Finally, the stem was revisioned.

**Fig. 4:**
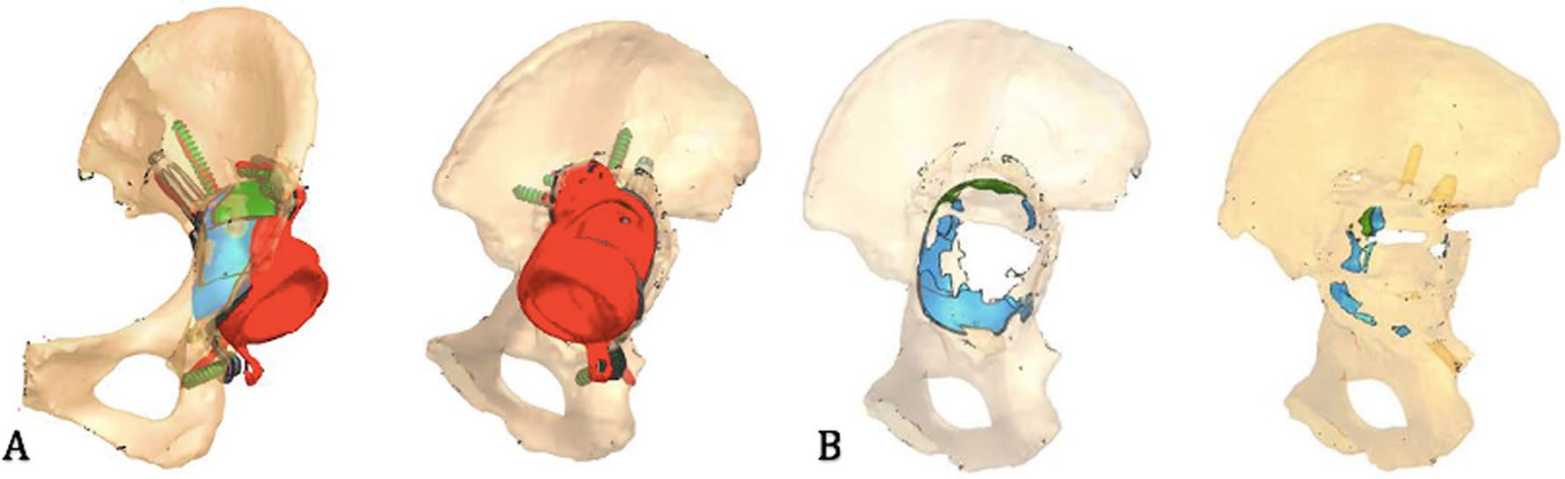
**A** Planned implant (gray/blue/green) versus achieved position (red); **B** trabecular structure in contact with the bone planned versus achieved

The achieved INCL position of the implant was 44.7° compared to the 40° planned, while the achieved AV was 14.8° compared to the 15° planned. The percentage between the planned and achieved implant surface in contact with the bone was 27.6 %, as the planning resulted in a trabecular surface of 2252 mm^2^, while the achieved one was only 623 mm^2^ (Fig. 4).

The preoperative planning estimated four iliac screws, together with two ischial screws and one iliac stem. As regards the screw’s number, five out of the six screws were used as planned. All four iliac screws were oriented as the planned direction, three out of the four had the same length as planned, and one screw turned out to be shorter than the planning. Concerning the two ischial screws positioning, one ischial screw turned out to be oriented distally, and longer than the planning, and another one was not used (Additional file 2). Moreover, the iliac stem was positioned with a discrepancy of 7.7° compared to the planning (Additional file 3).

### Case 3

**Fig. 5:**
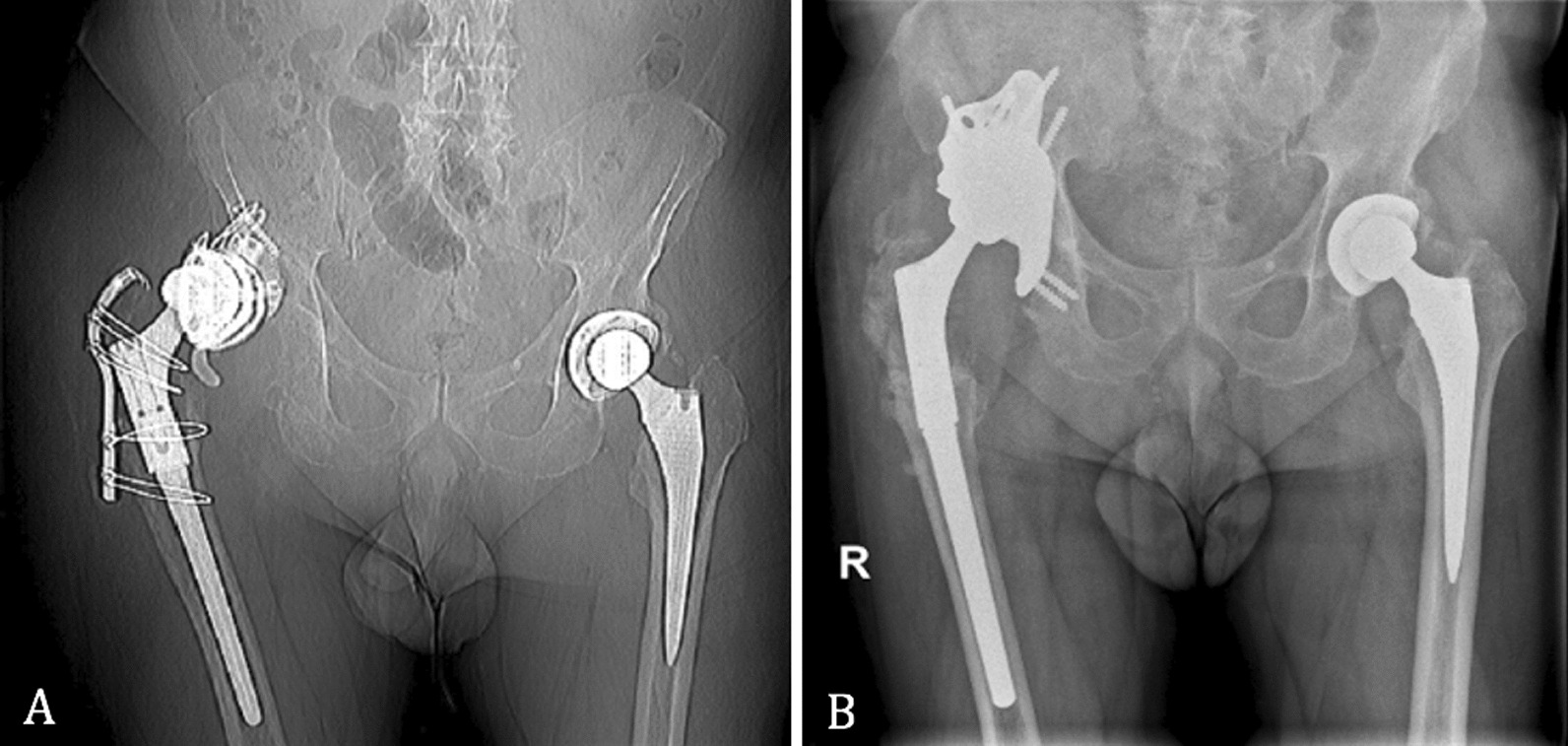
**A** Pre- and **B** post-custom-made implant

The third patient was male, BMI of 25.4 kg/m^2^, with a low activity level. A THA to treat osteoarthritis of the right hip had been performed, and subsequently revisioned for mobilization of the component. The preoperative analysis identified a Paprosky 3A acetabular defect. The custom-made implant was designed from a CT scan 6 months prior to the operation. Afterward, an acetabular custom-made implant through a posterolateral approach was ruled out for the failure of the previous revision implant (Fig. 5).

A double flange implant with eight iliac screws and two ischial screws was designed taking into consideration the poor bone quality (accentuated caudally), the massive acetabular erosion, and the CT scan quality leading to uncertainty in 3D reconstruction.

3D anatomical plastic sterile models were utilized to support the necessary comparison of the acetabular exposition and the corresponding prepared model. Homologous bone grafts in chips were used to fill the massive posterior, superior, and anterior wall defects. After the achievement of good stability with an acetabular phantom, the custom-made prosthesis was implanted. Finally, the stem was revisioned.

**Fig. 6:**
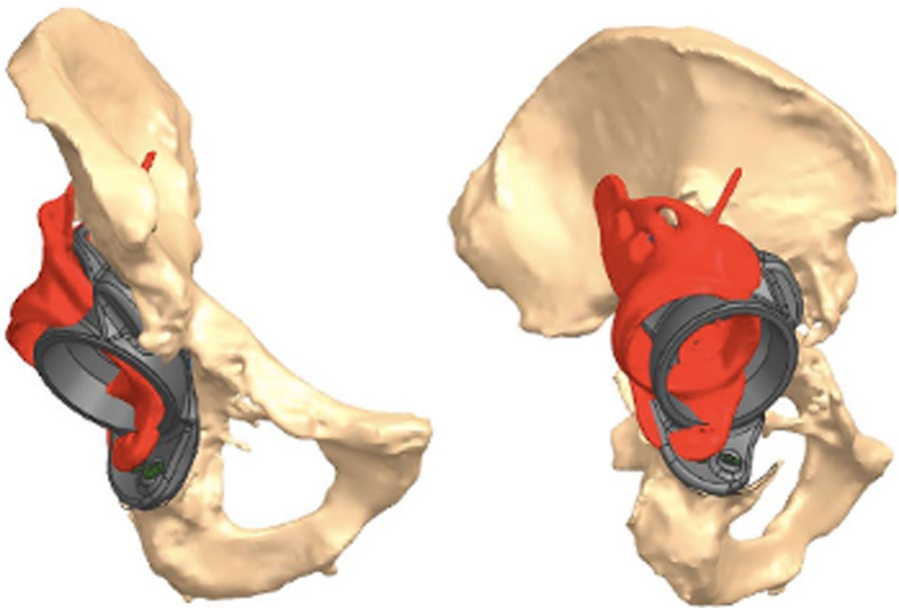
Planned implant (gray) versus achieved position (red)

**Fig. 7:**
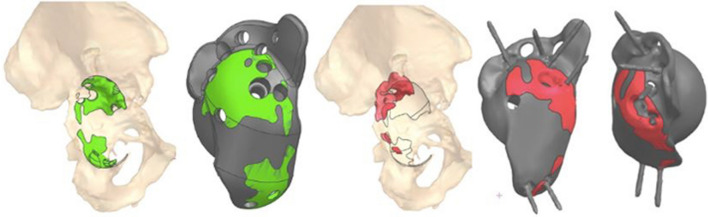
Trabecular structure in contact with the bone: planned (green) versus achieved (red)

The achieved INCL position of the implant was 34° compared to the 40° planned, while the achieved AV was 15° compared to the 20° planned (Fig. 6). The percentage between the planned and achieved implant surface in contact with the bone was 61.5%, as the planning resulted in a trabecular surface of 2330 mm^2^, while the achieved one was 1432 mm^2^ (Fig. 7).

As regards the number and position of the screws, four out of the eight iliac screws planned were positioned, as well as all the two ischial screws planned. Three out of the four iliac screws positioned have a good engagement with the native bone, while the remaining screw seems to not have contact with the bone. Finally, the two ischial screws have excellent contact with the native bone (Additional file 4).

## Discussion

The current case series revealed promising accuracy of positioning of acetabular custom-made devices compared to the planned implant designed on preoperative 3D CT.

The interest and application of custom-made devices to treat massive acetabular bone loss in rTHA are rising [27]. A comparative analysis of rTHA suggests a higher incidence of aseptic loosening requiring re-revisions in standard acetabular prosthesis compared to custom-made implants further vindicating the use of those implants [28]. However, this procedure presents several risk factors for treatment failure to be considered, such as PJI, additional revision of femoral component, rheumatoid disease, and elevated preoperative CRP [29]. Additionally, restoring a correct acetabular orientation in Paprosky type 3 rTHA could be difficult due to the loss of normal bony landmarks [30].

Accurate planning and 3D anatomical models could help in assessing acetabular bone deficiencies reliably in rTHA [31]. Restoring the correct CoR and acetabular position is an important goal to avoid wear of the prosthesis components, risk of aseptic loosening, and risk of malfunction of the abductor muscles. However, there is no clear evidence of how a change in CoR is clinically relevant. In fact, a study supported an increased risk of aseptic cup revision for every 1 mm medial or lateral distance away from the native hip center to the prosthetic head center [32]. Moreover, a lateral CoR deviation over 18 mm leads to an increased risk of aseptic loosening [33]. Instead, other authors claimed that wear was related to the inclination of the cup but not to a change in the CoR [34]. Finally, a superior placement of a hip center, more than 15 mm above the true hip center, delayed the recovery of the abductor muscle moment after THA [35].

According to the literature, malpositioning of the implant compared to the planned was defined as a deviation of more than 10° for inclination/anteversion and more than 5 mm for CoR [21]. Several articles describing the accuracy in positioning of traditional implants in rTHA were published [36]. However, in the literature only a few studies examining custom-made implant positioning were available. The accuracy of the positioning of acetabular custom-made implant has been analyzed by matching pre- and postoperative CT scans to compare CoR, AV, and INCL planned versus achieved [21–25]. The current case series showed positive results, with only a malpositioning in the third case due to the posterior shift of the CoR of the implant of more than 10 mm. This inaccuracy could be caused by a difficult intra-operative orientation compromised due to the deficiency of anatomical landmarks and the lack of patient-specific instrumentation. A possible edge for more accurate positioning of the implant is patient-specific instrumentation and drill guides [37]. However, the other CoR vectors considered in the third patient and all vectors in the other two cases fall within the cut-offs. Moreover, all the cases were positioned with a difference of less than 10° of AV and INCL compared to the planning. Those results are aligned with the current literature, where a mean high percentage of good positioning in the available articles was obtained (Table 2).

**Table 2:** Studies comparing planned and achieved positioning of custom-made with CT scans

References	No of patients	CoR shift			∆Inclination	∆Anteversion
		∆Cranial shift	∆Posterior shift	∆Lateral shift		
Baauw et al. [21]	16	+ 3.2 mm (− 1/+ 17.9)	+ 0.6 mm (− 1.8/+ 7)	+ 1.3 mm (− 1.3/+ 5)	+ 3° (− 2/17)	− 4.1° (− 16/+ 5)
Weber et al. [22]	11	+ 0.4 mm (− 5/+ 5.2)	+ 1.1 mm (− 6.5/+ 5)	+ 0.3 mm (− 5/+ 7.5)	3.6° (− 2/+ 7.5)	− 1.2° (− 9.5/+ 15.5)
Durand-Hill et al. [23]	20	+ 0.4 mm (− 6/+ 19)	+ 0.2 mm (− 7/+ 9)	+ 0.45 mm (− 10/+ 10)	0.75° (− 11/12)	+ 2° (− 13/19)
Zampelis and Fivlik [24]	10	1.1 mm (IQR − 1.6/+ 2.8)	0.5 mm (IQR − 0.7/+ 2.9)	0.6 mm (IQR + 0.1/+ 1.8)	3.6° (− 10.7/+ 11.2)	− 2.8° (− 12/+ 5.7)

*V* anteversion, *CoR* center of rotation, *INCL* inclination, *IQR* interquartile range, *N* number

Baauw et al. [21] described a malposition for cranialization and inclination due to an acetabular intra-operative fracture, and three cases of a malposition in AV. Weber et al. reported four malpositions in restoring CoR, while Wessling et al. had three cases in AV and two in INCL [22, 25]. Zampelis et al. [24] did not describe any cases over 10 mm of displacement from the planned CoR, two malpositions for INCL, and one for AV. Finally, Durand et al. described ten cases of displacement in CoR, two in INCL, and four in AV [23].

Furthermore, in this case series, a difficulty in the positioning of the planned screws was observed. A total of 15 screws compared to the 22 planned screws were positioned, whose eight screws were placed longer, shorter, or not completely in contact with the bone. Similar discrepancies were reported in the literature. In the case series of Wessling et al., a mean of four additive screws were implanted [25]. Moreover, in the work of Baauw et al., a difference from the planned screws number in 11 cases of 16 was observed [21].

The percentage of acetabular custom-made surface in contact with the bone had not yet been described. The smaller bone contact area of the implant could have been caused by several factors. The interposition of soft tissue on the surface of the bony structure where the acetabular custom-made implant was placed could cause errors in the bone-to-implant fitting [38]. Another possible cause was the inadequate preparation of the acetabular cavity, as a result of the lack of patient-specific instrumentations and drill guides [37]. Also, the time necessary for designing and manufacturing the custom-made implant, from preoperatory CT to final surgery, could have played a role to obtain a high percentage of the implant surface with the bone, as acetabular defects and bony voids may change over time. In fact, even in the case of optimal positioning, the second implant reached only 27% of contact, which may be caused by the large interval of 24 months from preoperatory CT to surgery. Nevertheless, results of this case series reveal primary stability of the implant also with a low percentage of contact compared to the planned. However, a medium/longer-term follow-up is needed to declare stability able to last over time and to prevent the risk of aseptic loosening with a low rate of contact of the implant with the bone.

The current study presents several limitations. First of all, a retrospective descriptive study design was used. Secondly, the case series is very limited by numbers with only three cases considered, thus impairing the possibility to perform a statistical analysis. Moreover, no clinical outcome was investigated. Consequently, a clinical evaluation taking into account the achieved accuracy of the implant was not possible.

These three cases that underwent surgery with the 3D printed customized acetabular implants presented in the current study showed satisfactory results in accuracy and excellent primary stability. However, further studies with a larger number of patients considering clinical outcomes at long-term follow-up are needed to confirm these results and to investigate the association between the improvement of accuracy and the rise of clinical outcomes and stability that lasts over time.

## Conclusion

The current case series revealed promising accuracy in the positioning of custom-made acetabular prosthesis comparing the planned implant in preoperative CT scans with postoperative CT. However, further articles including a larger pool of patients are requested to confirm these preliminary results.

### Supplementary Information

**Additional file 1.** Implant superimposition for screws usage, planned (green) versus post-op (orange).

**Additional file 2.** Implant superimposition for screws usage, planned (green) versus post-op (red).

**Additional file 3.** Implant superimposition for stem angular mismatch, planned (gray axis) versus achieved (green axis).

**Additional file 4.** View of the planned screw fixation (green) versus view of the implanted screws engagement with the bone (red).
