# Supplementary figures and images for: Custom-made implants for massive acetabular bone loss: accuracy with CT assessment
Source: J Orthop Surg Res. 2023 Sep 30;18:742. doi: 10.1186/s13018-023-04230-5 (PMC10544156; doi:10.1186/s13018-023-04230-5)

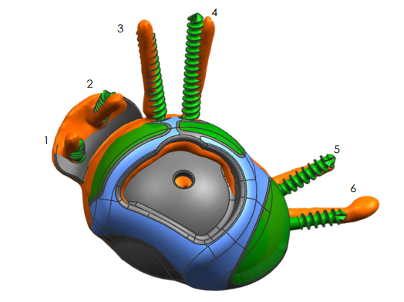

Supplement: Supplementary file 1 — Additional file 1. Implant superimposition for screws usage, planned (green) versus post-op (orange). [file 13018_2023_4230_MOESM1_ESM.tiff]

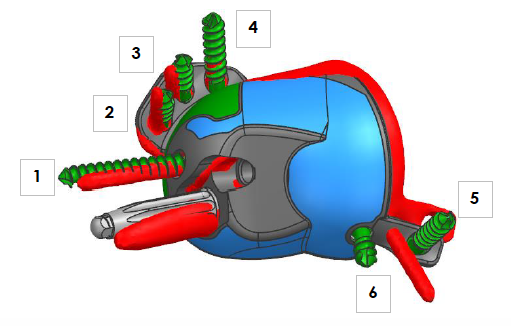

Supplement: Supplementary file 2 — Additional file 2. Implant superimposition for screws usage, planned (green) versus post-op (red). [file 13018_2023_4230_MOESM2_ESM.tiff]

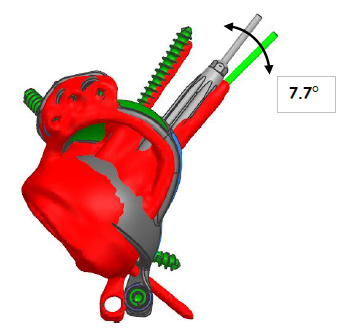

Supplement: Supplementary file 3 — Additional file 3. Implant superimposition for stem angular mismatch, planned (gray axis) versus achieved (green axis). [file 13018_2023_4230_MOESM3_ESM.tiff]

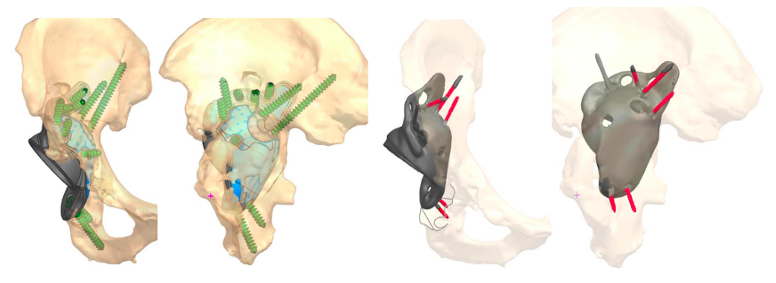

Supplement: Supplementary file 4 — Additional file 4. View of the planned screw fixation (green) versus view of the implanted screws engagement with the bone (red). [file 13018_2023_4230_MOESM4_ESM.tiff]
